# Renal Denervation Improves the Baroreflex and GABA System in Chronic Kidney Disease-induced Hypertension

**DOI:** 10.1038/srep38447

**Published:** 2016-12-05

**Authors:** Hsin-Hung Chen, Pei-Wen Cheng, Wen-Yu Ho, Pei-Jung Lu, Chi-Cheng Lai, Yang-Ming Tseng, Hua-Chang Fang, Gwo-Ching Sun, Michael Hsiao, Chun-Peng Liu, Ching-Jiunn Tseng

**Affiliations:** 1 Department of Medical Education and Research, Kaohsiung Veterans General Hospital, Kaohsiung, Taiwan; 2 Institute of Clinical Medicine, National Yang-Ming University, Taipei, Taiwan; 3 Yuh-Ing Junior College of Health Care & Management, Kaohsiung, Taiwan; 4 Division of General Internal Medicine, Department of Internal Medicine, Kaohsiung Medical University Hospital, Kaohsiung Medical University, Kaohsiung, Taiwan; 5 Graduate Institute of Clinical Medicine, National Cheng-Kung University, Tainan, Taiwan; 6 Cardiovascular Center, Kaohsiung Veterans General Hospital, Kaohsiung, Taiwan; 7 Department of Pathology and Laboratory Medicine, Kaohsiung Veterans General Hospital, Kaohsiung, Taiwan; 8 Division of Nephrology, Kaohsiung Veterans General Hospital, Kaohsiung, Taiwan; 9 Department of Anesthesiology, Kaohsiung Medical University Hospital, Kaohsiung Medical University, Kaohsiung, Taiwan; 10 Genomics Research Center, Academia Sinica, Taipei, Taiwan; 11 Department of Administration, Kaohsiung Veterans General Hospital, Kaohsiung, Taiwan; 12 Section of Cardiology, Department of Medcine, Tri-Service General Hospital, National Defense Medical Center, Taipei, Taiwan; 13 Department of Medical Research, China Medical University Hospital, China Medical University, Taichung, Taiwan; 14 Institute of Biomedical Sciences, National Sun Yat-sen University, Kaohsiung, Taiwan

## Abstract

Hypertensive rats with chronic kidney disease (CKD) exhibit enhanced gamma-aminobutyric acid (GABA)_B_ receptor function and regulation within the nucleus tractus solitarii (NTS). For CKD with hypertension, renal denervation (RD) interrupts the afferent renal sympathetic nerves, which are connecting to the NTS. The objective of the present study was to investigate how RD improves CKD-induced hypertension. Rats underwent 5/6 nephrectomy for 8 weeks, which induced CKD and hypertension. RD was induced by applying phenol to surround the renal artery in CKD. RD improved blood pressure (BP) by lowering sympathetic nerve activity and markedly restored the baroreflex response in CKD. The GABA_B_ receptor expression was increased in the NTS of CKD; moreover, the central GABA levels were reduced in the cerebrospinal fluid, and the peripheral GABA levels were increased in the serum. RD restored the glutamic acid decarboxylase activity in the NTS in CKD, similar to the effect observed for central treatment with baclofen, and the systemic administration of gabapentin reduced BP. RD slightly improved renal function and cardiac load in CKD. RD may improve CKD-induced hypertension by modulating the baroreflex response, improving GABA system dysfunction and preventing the development and reducing the severity of cardiorenal syndrome type 4 in CKD rats.

Hypertension occurs in more than 80% of patients with chronic kidney disease (CKD)^1^. Patients with CKD have a higher risk of developing cardiovascular diseases than the general population^2^,^3^. Multiple guidelines discuss the importance of lowering blood pressure (BP) to slow the progression of renal disease and reduce cardiovascular morbidity and mortality^4^. However, to achieve and maintain adequate BP control, most patients with CKD require multiple antihypertensive agents. Despite the increasing prevalence of CKD-induced hypertension, the awareness of hypertension among individuals with CKD remains suboptimal, and the rates of BP control remain poor^5^. Recently, many studies have provided evidence that renal denervation (RD) has beneficial effects in patients with CKD-induced hypertension^6^. However, RD improves CKD progression through unknown mechanisms.

Several forms of renal injury can induce the activation of sensory afferent signals. The afferent impulses from a kidney injured by phenol injection in the lower pole of one kidney increase BP^7^. Renal afferent fibers transmit information to sympathetic and parasympathetic nerves, and this information converges at the nucleus tractus solitarii (NTS), which is the primary site of BP and sympathetic nerve activity (SNA) modulation^8^,^9^. The NTS is the site where afferent fibers arising from the arterial and cardiopulmonary baroreceptors make the first central synapses. Experimental lesions of the NTS lead to a loss of baroreflex control of BP and sympathetic activation, and cause severe hypertension in animals^10^. Durgam *et al*. showed that hypertensive rats with CKD exhibit enhanced gamma-aminobutyric acid (GABA)_B_ receptor function and regulation within the NTS^11^. The region of the NTS where the baroreceptor afferents terminate contains a high density of both GABA_A_ and GABA_B_ receptors. Additionally, brain GABA release, uptake and degradation are decreased by human uremia^12^. The injection of GABA_B_ receptor agonists within the NTS produces an inhibition of NTS neurons that reduces the baroreflex and bradycardia and increases BP in acute and chronic hypertension^13^, thus enhancing presynaptic inhibition^14^. However, the role of GABA system dysfunction in the NTS in CKD-induced hypertension has not been defined.

Martin *et al*. showed that CKD is associated with increased cardiovascular morbidity and mortality, and this association further supports the concept of a kidney-heart connection^15^. Cardiorenal syndrome (CRS) type 4 or chronic renocardiac syndrome is characterized by primary CKD, leading to an impairment of cardiac function, with left ventricular hypertrophy (LVH), diastolic dysfunction, and/or an increased risk of adverse cardiovascular events^16^. RD reduces LVH and improves cardiac function^17^ and thus may provide a new approach for the treatment of CKD with hypertension^18^. In the current study, we hypothesized that RD improves GABA system dysfunction through the NTS, and leads to decreased BP and lowered sympathetic activity of hypertensive rats with CKD; these changes further attenuate CRS type 4. This study suggested a possible normalization effect on the GABA system in CKD-induced hypertension via baroreflex-mediated regulation of the NTS, and this effect prevented the development of CRS type 4.

## Results

### RD reduces BP by improving SNA and restores the baroreflex response in CKD

**Figure 1:**
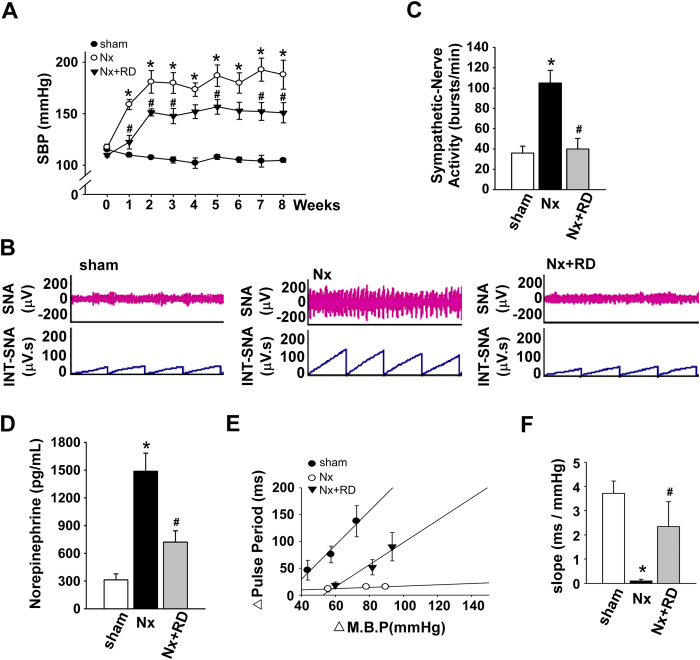
Renal denervation attenuates hypertension and sympathetic hyperactivity and restores the baroreflex response in CKD-induced hypertension. (**A**) Time course of SBP in each group over 8 weeks. (**B**) The representative traces show the baseline renal SNA in each group at week 8. (**C**) The renal SNA was measured in each group after 8 weeks. (**D**) The change in the norepinephrine serum level was measured in each group after 8 weeks. (**E**) The points and vertical bars represent the increases in the pulse period of the peak bradycardic response in response to the suppressive effects of different doses of phenylephrine. The lines connecting the points were obtained using linear regression analysis, which yielded the slopes of each group. (**F**), Effects of NTS on the baroreflex response (slope) to phenylephrine in each group. The data are shown as the means ± SEM (n = 6–8). *P < 0.05 versus the sham group. ^#^*P* < 0.05 versus the Nx group.

To evaluate the modulatory effect of RD on the BP of CKD rats, we investigated the therapeutic effects of RD on the BP and the peripheral nervous system. Figure 1A shows that the hypertensive response of the 5/6 nephrectomized (Nx) rats that underwent RD was markedly reduced. At week 1 after RD, the systolic blood pressure (SBP) was markedly decreased (158 ± 5 versus 122 ± 6 mm Hg, *P* < 0.05). A continued decrease in SBP was observed 8 weeks after RD (187 ± 14 versus 155 ± 9 mm Hg, *P* < 0.05). The SNA significantly increased (Fig. 1B) in the Nx groups at 8 weeks, and RD significantly attenuated the SNA compared with that in the Nx group (40 ± 10 versus 105 ± 12 bursts/min, *P* < 0.05, Fig. 1C). We observed that the norepinephrine serum levels were markedly increased (Fig. 1D) in the Nx groups at 8 weeks, and RD significantly lowered the norepinephrine levels in the CKD group.

We further investigated whether RD improves the CNS regulation of BP, which occurs via the NTS-regulated baroreflex response, in hypertensive rats with CKD. Intravenous injection of phenylephrine (10 to 30 μg/kg IV) increased the BP and pulse period in a dose-dependent manner (Fig. 1E). Baroreflex responses were elicited by increasing doses of phenylephrine in the Nx and Nx+RD groups; these responses were different from those found in the sham group. The baroreflex responses were abolished after 5/6 nephrectomy (Fig. 1F). However, in the Nx+RD group, the baroreflex responses partially recovered.

### RD reverses increased GABA_B_ receptor expression and normalizes GABA levels in CKD

**Figure 2:**
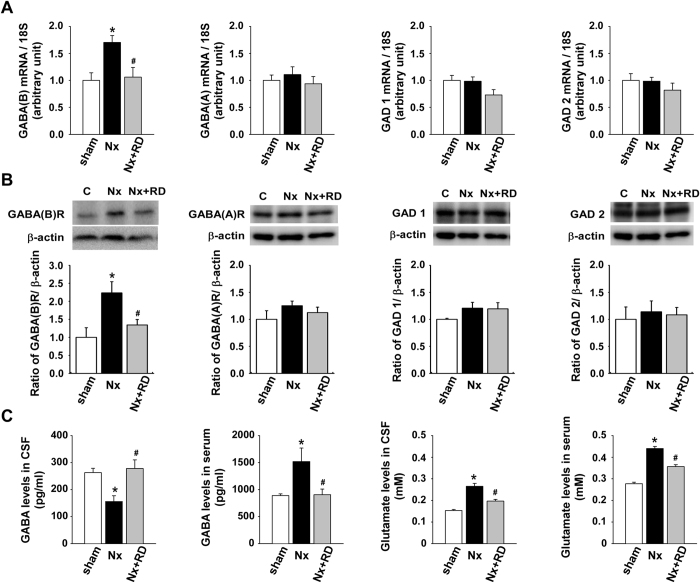
The expression levels of the GABA_B_ receptor are increased and GABA levels are decreased in CKD-induced hypertension. (**A**) The mRNA levels of the GABA receptors and enzymes in each group were determined using real-time PCR. (**B**) The protein levels of the GABA receptors and enzymes in each group were determined using western blotting. (**C**) The GABA and glutamate levels in the CSF and serum in each group were determined using an ELISA kit. The data are shown as the means ± SEM (n = 4–8). *P < 0.05 versus the sham group. ^**#**^*P* < 0.05 versus the Nx group.

We examined the expression of components of the GABA system in the central and peripheral nervous system in each group. The GABA_B_ receptor mRNA levels in the NTS were increased in the Nx group, and RD reversed this effect (Fig. 2A). There were no differences in the levels of GABA_A_ receptor, glutamate decarboxylase (GAD) 1 and GAD 2. The immunoblotting analysis revealed similar results (Fig. 2B). To test whether the increased GABA_B_ receptor expression was due to a feedback response in the Nx group, we further measured the central and peripheral GABA and glutamate concentrations. In the cerebrospinal fluid (CSF), the GABA levels were decreased (263.07 ± 15.89 versus 156.12 ± 21.07 pg/ml *P* < 0.05) and the glutamate levels were increased (0.153 ± 0.005 versus 0.265 ± 0.014 nM *P* < 0.05) in the Nx group compared with those in the sham group; RD reversed these changes (Fig. 2C). In the serum, the GABA (890.07 ± 30.88 versus 1519.77 ± 249.43 pg/ml, *P* < 0.05) and glutamate levels were increased (0.278 ± 0.006 versus 0.440 ± 0.009 nM, *P* < 0.05) in the Nx group compared with those in the sham group; RD reversed these changes (Fig. 2C). Our results indicated that the decrease in the endogenous GABA levels in the CNS may lead to increased expression of the GABA_B_ receptor in the NTS during CKD.

### RD improves GAD activity and reverses GABA dysfunction in CKD with hypertension

**Figure 3:**
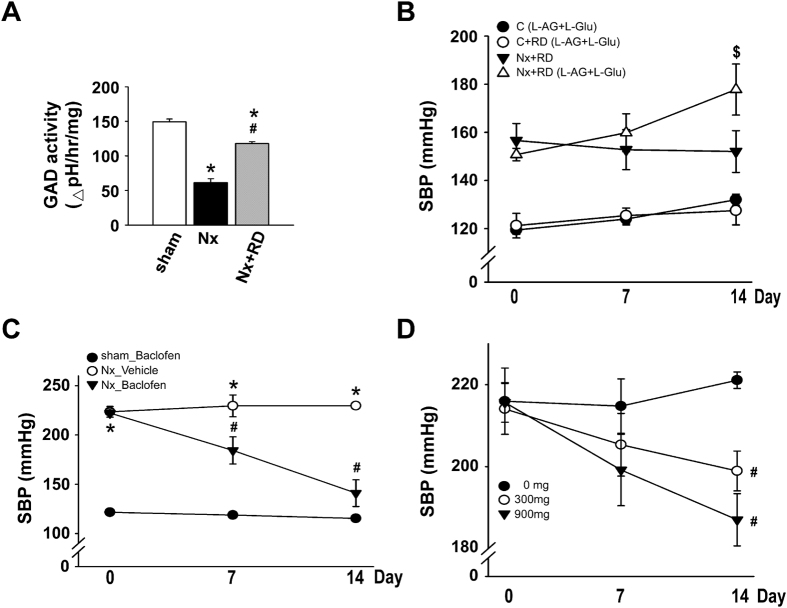
Renal denervation and GABA drugs restore GAD activity, which contributes to BP regulation. (**A**), GAD activity was measured using the optical density of the pH variations in the NTS at 600 nm. (**B**) The ICV injection of L-AG with L-Glu via a minipump for 2 weeks promoted SBP changes in the Nx+RD group. (**C**) The ICV injection of baclofen via a minipump for 2 weeks promoted SBP changes in the hypertensive rats. (**D**) Different doses of oral gabapentin promoted SBP changes in the Nx group for 2 weeks. The data are shown as the means ± SEM (n = 4–6). *P < 0.05 versus the sham group. ^**#**^*P* < 0.05 versus the Nx group. ^**$**^*P* < 0.05 versus the Nx+RD group.

Our data showed that the GABA system is unbalanced in the Nx group. However, it is not clear whether the down-regulated GAD activity decreased the GABA levels in the CNS. First, we analyzed the GAD activity in the NTS of each group. The GAD activity was inhibited in the Nx group compared with that in the sham group; conversely, this inhibition was reversed in the Nx+RD group (Fig. 3A). In the Nx group, the GABA levels decreased and the glutamate levels increased in the CSF, and the GAD activity decreased in the NTS (Figs 2C and 3A). Therefore, we used intracerebroventricular (ICV) injection of an L-allylglycine (L-AG) and L-glutamate (L-Glu) combination in non-nephrectomized rats to mimic the central effect of the Nx group. ICV injections of the L-AG and L-Glu combination elevated BP, but L-AG or L-Glu alone did not change the BP in non-nephrectomized rats (Supplementary Fig. 2). Next, we investigated whether the decrease in GABA levels and the increase in glutamate levels in the central of the Nx+RD groups would block the hypotensive reaction caused by renal denervation. In non-nephrectomized rats, ICV injections of L-AG and L-Glu combination showed similar effect on the BP in the denervated and non-denervated rats. Furthermore, ICV injections of L-AG and L-Glu combination elevated the BP in the Nx+RD group reversing the hypotensive effect of denervation (Fig. 3B). Moreover, we utilized an ICV injection of baclofen or oral administration of a GABA analog (gabapentin) to determine whether the decreased BP was associated with the increased GABA levels in CKD with hypertension. ICV baclofen and oral gabapentin markedly decreased the BP in the treated Nx group compared with that of the untreated Nx group (Fig. 3C,D). Our results indicated that treatments that improve defects in GAD activity may restore the GABA levels and decreased the BP in CKD-induced hypertension.

### RD improves chronic CRS type 4 in CKD

**Figure 4:**
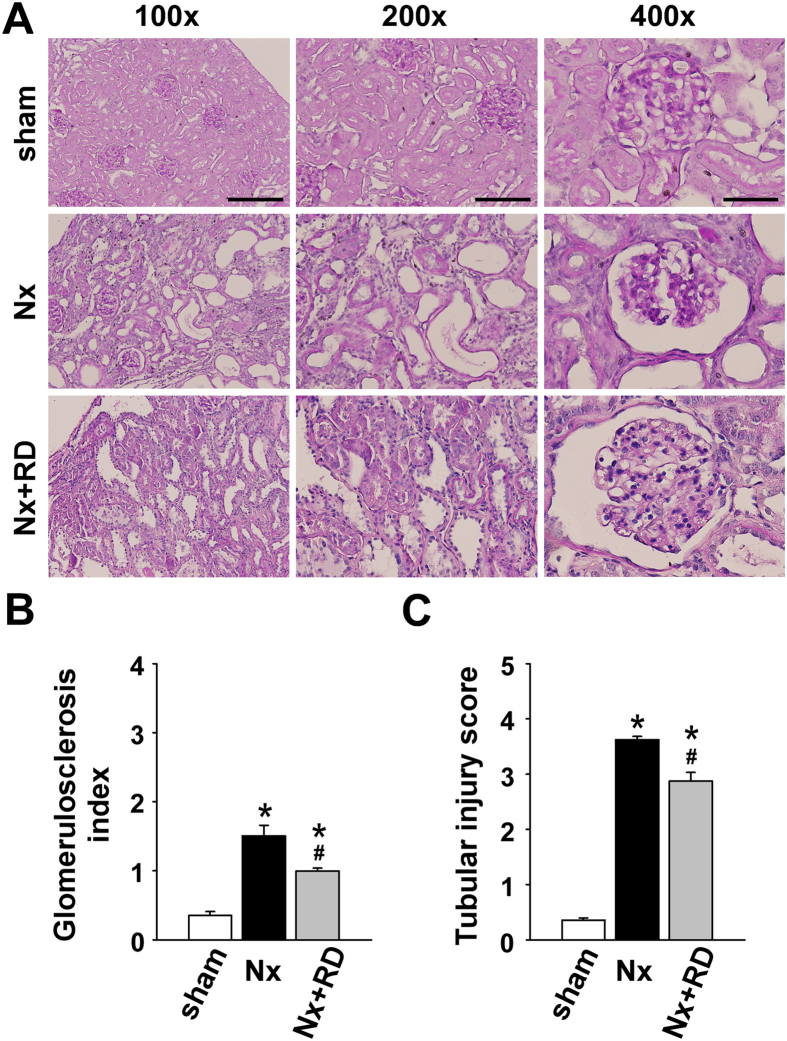
Renal denervation decreases renal damage in CKD. (**A**) Periodic acid-Schiff staining demonstrated the histopathological changes in the renal cortex in each group after 8 weeks. The effects on the (**B**) glomerulosclerosis index and (**C**) tubular injury score were assessed in each group. (Scale bars: 400 μm for the left column, 200 μm for the middle column, and 100 μm for the right column). Sham, sham-operated group; Nx, 5/6 nephrectomy group; RD, renal denervation group. The data are shown as the means ± SEM (n = 6–8). *P < 0.05 versus the sham group. ^#^*P* < 0.05 versus the Nx group.

We further investigated the therapeutic effects of RD on the kidney and heart function in CKD. Periodic acid-Schiff (PAS) staining demonstrated that RD slightly attenuated the glomerulosclerosis and tubular injury compared with that in the Nx group (Fig. 4). Additionally, RD also restored the serum BUN and creatinine and urinary protein and creatinine levels (Table 1). Nx decreased the urinary sodium and potassium levels compared relative to those in the sham group; however, the urinary sodium and potassium levels in the Nx+RD group were not significantly different from those in the Nx group. In the present study, we also obtained a complete blood count in which red blood cells (RBCs), hemoglobin (Hb) and hematocrit (Hct) decreased in Nx groups, whereas the Nx+RD group restored these effects (Supplementary Table S1).

**Figure 5:**
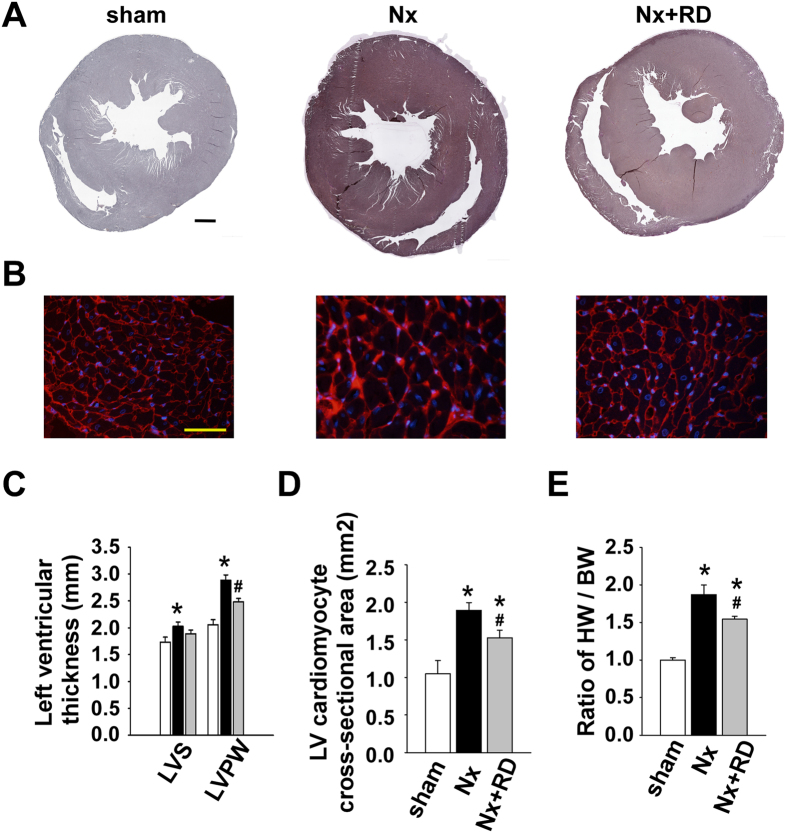
Renal denervation attenuates left ventricular hypertrophy in CKD. (**A**,**B**) Representative gross pathology section from the cardiac mid-chamber (Elastic Van Gieson stain, scale bar, 1 mm) and wheat germ agglutinin (WGA)-stained sections from the mid-chamber free wall (scale bar, 100 μm) in each group at 8 weeks. (**C**) The Nx group exhibited a significantly increased thickness of the left ventricular septum and posterior wall; however, this effect was reversed in the Nx+RD group. (**D**) The Nx group exhibited a significant increase in the cross-sectional surface area of individual cardiomyocytes; however, this effect was reversed in the Nx+RD group. (**E**) The Nx group exhibited a significant increase in the heart weight to body weight ratio by week 8; however, this effect was reversed in the Nx+RD group. The data are shown as the means ± SEM (n = 6–8). n = 100 cells per group for the WGA analysis. *P < 0.05 versus the sham group. ^**#**^*P* < 0.05 versus the Nx group.

We evaluated wheter 5/6 nephrectomy increased the cardiac load and whether RD reversed the effect. Representative gross pathology tissue sections demonstrated Nx-mediated LVH characterized by thickened ventricular walls and reduced internal chamber diameter, whereas RD attenuated these effects (Fig. 5A,C). Microscopic analysis of cardiomyocytes stained with fluorochrome-labeled wheat germ agglutinin (WGA) revealed a significant increase in cross-sectional surface area in the Nx group and indicated hypertrophic growth of individual myocytes (Fig. 5B,D). RD slightly ameliorated the LVH in the Nx+RD group. The absolute values of heart weight and body weight showed similar effects (Supplementary Table S2).

**Figure 6:**
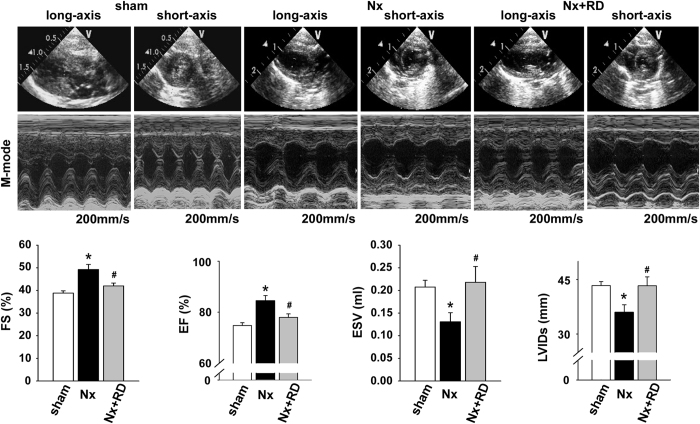
Echocardiographic examinations of CKD. The echocardiographic assessments show the short-axis view and M-mode for each group of rats. The Nx group exhibited a significant increase in the fractional shortening (FS) and ejection fraction (EF); however, these effects were reversed in the Nx+RD group. The Nx group exhibited a significant decrease in the left ventricular internal diameter in systole (LVIDs) and end systolic volume (ESV); however, these effects were reversed in the Nx+RD group. The data are shown as the means ± SEM (n = 6–10). *P < 0.05 versus the sham group. ^**#**^*P* < 0.05 versus the Nx group.

We also examined echocardiography data that revealed increases in the fractional shortening and ejection fraction in the Nx groups, but the left ventricular internal diameter in systole and the end systolic volume was significantly decreased, and these effects were reversed by RD (Fig. 6). RD slightly improved the changes in interventricular septum, left ventricular posterior wall, cardiac output, heart rate, and R-R interval (Supplementary Table S3).

## Discussion

The goal of this study was to elucidate the mechanisms involved in chronic 5/6 nephrectomy-induced hypertension and how RD reduces hypertension in a model of CKD-induced hypertension. We found that RD attenuated sympathetic hyperactivity, lowered elevated blood pressure, improved abnormal renal and cardiac structure and function, and improved impaired baroreflex responses, along with the normalization of altered GABA receptor and GABA levels in the NTS of CKD rats.

CKD is attributed to hypertension, and its incidence continues to rise worldwide. Sympathetic hyperactivity signifies a hypertensive state and participates in the initiation, maintenance, and progression of elevated BP^19^. However, hypertension is the outcome of a primary shift of the CNS-mean arterial pressure set-point to a higher operating pressure, which results in increased SNA^20^. Recently, therapeutic baroreceptor stimulation^21^ and renal sympathetic nerve ablation^22^ have both been shown to reduce central sympathetic tone. Our study indicated that RD may attenuate sympathetic hyperactivity and improve the baroreflex through the NTS in CKD-induced hypertension. The NTS is essential for coordinating the arterial baroreflex control of BP. In CKD rats, we discovered GAD activity dysfunction in the NTS, and further observed an increase in the glutamate level and a decrease in the GABA level in the CSF. Therefore, we used ICV injections of an L-AG and L-Glu combination in Nx+RD rats, which led to a recovery of the hypotensive reaction. Recent evidence has suggested that renal denervation ablates renal afferent nerve signals and reduces sympathetic hyperactivity. The baroreflex was impaired in the nephrectomized groups, but the non-nephrectomized rats had a normal baroreflex response. We suggest that renal denervation does not affect efferent signals, and as a result, ICV injections of the L-AG and L-Glu combination elevated BP. Moreover, the hypertensive effect of L-AG and L-Glu was not changed by denervation in the non-nephrectomized rats. ICV injections of L-AG and L-Glu abolished the hypotensive effect of denervation in nephrectomized rat. The protective effect of denervation in CKD is derived from the blockade of adverse afferent effect from the kidney into GABA system in the NTS.

Autopsies of 10 patients with end-stage renal disease and dialysis encephalopathy revealed a reduction in the GABA levels in many areas of their brains^23^. Conversely, oral administration of GABA and GABA-containing products attenuated hypertension in spontaneously hypertensive rats, Izumo strain (SHR/Izm)^24^. Chronic dietary administration of GABA inhibited the development of hypertension due to the GABAergic system effects in SHR, but there were no significant differences in denervated SHR^25^. Additionally, normalizing the GABA equilibrium potential may have some utility in treating Na^+^-dependent hypertension^26^. The basal outflow of GABA and glutamate was increased, but the potassium-stimulated GABA and glutamate release were less sensitive to Ca^2+^ depletion in the medial preoptic area (MPOA) of the uremic animals^27^. Glutamate suppresses GABA release via presynaptic metabotropic glutamate receptors in baroreceptor neurons^28^. We found that the glutamate levels increased, and GAD dysfunction led to reduced GABA levels and increased GABA_B_ receptor expression in the feedback loop of the NTS in CKD. In the present study, CKD-induced hypertension is mediated in part by activation of renal afferent nerves that cause an abnormal GABA system in the NTS, thus leading to abnormal baroreceptor sympathetic hyperactivity. Additionally, our study showed that the serum GABA levels were also elevated. The GABAergic controlling system may also play an important role in the peripheral system, including the kidney. The existence of a renal GABAergic system with an autocrine/paracrine mechanism has been suggested.

RD could be used in future applications for hypertension and related comorbidities and outcomes, such as metabolic disease, CKD, and cardiovascular disease^29^. However, a study of patients with psychotic disorders showed that 39.9% had prehypertension and 10.0% had hypertension, but only 3.6% received antihypertensive drugs^30^. Indeed, it is well established that individuals with serious mental illnesses have high cardiovascular morbidity and mortality. In our study, oral gabapentin decreased BP in a dose-dependent manner in CKD-induced hypertension. Thus, gabapentin may exhibit multiple therapeutic benefits in patients with hypertension and psychotic disorders. Gabapentin treatment for 4 weeks showed an improvement of 82% of the symptom severity of restless legs syndrome, which may be associated with cardiovascular mortality and survival in the uremic patients^31^,^32^. We look forward to the future, as gabapentin could have the multiple therapeutic benefits in patients with hypertension and psychotic disorders. Alternatively, our study found that ICV baclofen administration markedly decreased BP in CKD-induced hypertension; however, this treatment route is difficult to achieve in clinical settings. Therefore, RD may become another potential treatment approach in patients with psychotic disorders and hypertension.

Previously, bilateral nephrectomy was used to reduce the BP and sympathetic hyperactivity in patients receiving hemodialysis with CKD^33^. Recently, catheter-based RD to treat patients with CKD was shown to inhibit sympathoexcitatory reflexes, and the interruption of these afferent reflexes led to a reduction in systemic sympathetic tone^6^. Other potential effects of RD have been reported, including improvements in LVH^34^,^35^, an antiarrhythmic effect including atrial fibrillation^36^, and myocardial reperfusion injury^37^. Our study further showed that RD ameliorated the baroreflex and partially improved the renal function and LVH. RD ablates renal nerve afferent signals and reduces sympathetic hyperactivity. Furthermore, RD inhibits the elevation of BP and lowers kidney pressure, thus preventing further impairments associated with cardiorenal syndrome. Although an large clinical trial in the general population of resistant hypertensives failed to detect the protective effect of RD^38^, there still exist some evidences that implicates the potential benefit of RD in some specific conditions, including CKD. Additionally, CKD-specific risk factors such as anemia and hypertension, have been demonstrated to be closely associated with cardiovascular pathology and mortality^39^. Chronic anemia has also been shown to cause LVH^40^. Interestingly, our study demonstrated that RD could ameliorate CKD-caused anemia and further reduce LVH. The available data do not include information on the possible risks of high-dose iron management and high ferritin levels, and the limitations of intravenous iron therapy^41^. Indeed, several erythropoiesis-stimulating agents, such as iron, hepcidin, and ferroportin, still produce treatment-related adverse outcomes^42^; our study suggested that RD may become another beneficial therapeutic treatment. However, management of hypertension with CKD in patients is a marathon, not a sprint^43^.

**Figure 7:**
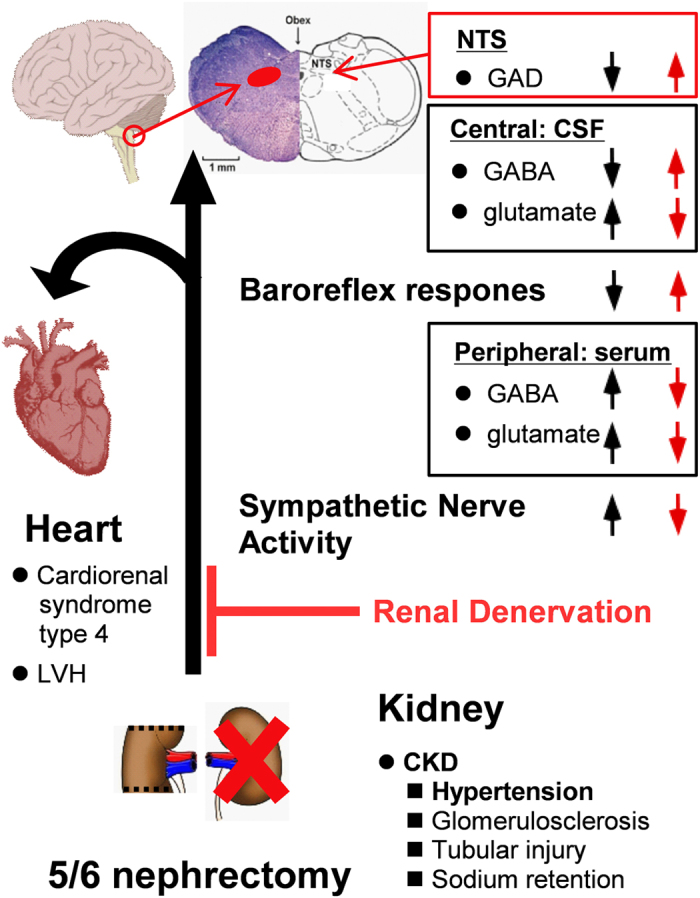
Schematic diagram summarizing the effects of renal denervation that improved CKD. CKD via 5/6 nephrectomy induced hypertension and LVH, enhanced the sympathoexcitatory reflexes, and impaired the baroreflex response. CKD activated the SNA, thus increasing serum GABA levels and serum and CSF glutamate levels. However, CKD decreased GABA levels. Additionally, the increased GABA_B_ receptor mRNA and protein levels in the NTS decreased GAD activity. RD inhibited SNA and improved the baroreflex. Thus, RD markedly reduced the BP and LVH symptoms and restored some renal function. Finally, RD normalized the function of the GABA system. The brain image was taken from (https://commons.wikimedia.org/wiki/File:Brain_stem_normal_human.svg), and the link to the license is (http://creativecommons.org/licenses/by/2.5/). The creators of the image are Patrick J. Lynch, medical illustrator, and C. Carl Jaffe, MD, cardiologist. The brainstem image was obtained from the RightsLink Printable License from this Agreement between Gwo-Ching Sun and John Wiley and Sons, which consisted of the license details and terms and conditions provided by John Wiley and Sons and Copyright Clearance Center. The heart drawing (https://commons.wikimedia.org/wiki/File:CoeurHumain.svg) is licensed under the Attribution-Share-Alike 3.0 Unported license. The license terms can be found at the following link: https://creativecommons.org/licenses/by-sa/3.0/.

In conclusion, this study indicates that renal sympathectomy may restore the baroreflex response and improve GABA function and further ameliorate renal dysfunction, LVH and the development of hypertension in CKD (Fig. 7). RD could reduce the levels of excitatory neurotransmitters and restore the levels of inhibitory neurotransmitters in CKD-induced hypertension and could further restore the balance in the autonomic nervous system. The reno-cerebral reflex reduced sympathetic hyperactivity, CRS type 4 and the development of hypertension following renal denervation. Our findings provide new insights into the CNS-mediated regulation of hypertension and demonstrate that renal denervation has pleiotropic systemic effects.

## Methods

### Experimental Chemicals

All experimental drugs were purchased from Sigma-Aldrich (St. Louis, MO, USA), unless otherwise indicated.

### Animal Care and Experiments

All animal experiments were obtained from the National Science Council Animal Facility (Taipei, Taiwan) and were performed according to a protocol approved by the Institutional Animal Care and Use Committees of Kaohsiung Veterans General Hospital. Eight-week-old male Wistar-Kyoto (WKY) rats were used for the 5/6 nephrectomy (Nx) experiments at 8 weeks. The animals were anesthetized with pentobarbital (50 mg/kg, i.p.), and divided into three groups as follows: (1) the sham group underwent sham surgery, which involved exteriorizing the left or right kidney and decapsulating it; (2) the Nx group^44^ underwent 2/3 nephrectomy of the left kidney and, 1 week later, total nephrectomy of the right kidney; and (3) the Nx+RD group^45^ consisted of rats from the Nx group in which the renal nerves were ablated by surrounding the renal artery with 20% phenol in ethanol.

Male WKY rats were ICV injected with a solution containing L-allylglycine (L-AG; 3.5 nmol/0.5 μl per hour, 2 weeks), an inhibitor of the GABA synthetic enzyme glutamate decarboxylase (GAD). Intracerebroventricular delivery was performed using an Alzet minipump (model 2002) filled with the L-AG solution, and the infusion rate was set at 5 μl per hour. GAD is an enzyme that catalyzes the decarboxylation of glutamate to GABA. The pump was then sutured under the skin in the nape of the neck, and the connector and cannula were cemented to the skull, as previously described^46^. After nephrectomy and/or denervation, an ICV injection was performed using a minipump for 2 weeks, and the blood pressure was measured in each group. They were divided into four groups as follows: the normotensive (WKY) rats were ICV injected with (1) an L-AG and L-Glu (2.3 nmol/0.5 μl per hour, 2 weeks) combination or (2) the L-AG and L-Glu combination with denervation. The Nx+RD groups were ICV injected with (3) vehicle and (4) the L-AG and L-Glu combination. Additionally, the Nx group was ICV injected using a minipump that was previously filled with a solution containing baclofen (0.1 nmol/0.5 μl per hour, 2 weeks), an agonist of the GABA_B_ receptor. Gabapentin is a structural analog of the inhibitory neurotransmitter GABA. Gabapentin (300 mg/kg/day or 900 mg/kg/day, 2 weeks) was administered orally to observe SBP changes in the Nx group.

### Blood Pressure Measurement

Using a previously described tail-cuff method (Model MK-2000 Storage Pressure Meter, Muromachi Kikai, Tokyo, Japan), the SBP was measured prior to the start of Nx and RD (day 0). Conscious animals were placed in an immobilizing device for 30 min. During this procedure, the reappearance of pulsation on the digital display of the BP cuff was detected by a pressure transducer and amplified and recorded as the SBP. During the measurement, ten individual readings were obtained in rapid succession. The highest and the lowest readings were eliminated, and the eight remaining readings were averaged.

During the ICV injection with baclofen and oral gabapentin treatment, blood pressure was measured at the start of the pharmacological intervention until the end of 2 weeks.

### Sympathetic Nerve Recordings and the Baroreflex Responses

The SNA was recorded as previously described^47^. The abdominal aortic nerve around the renal artery was identified through a peritoneal incision with the assistance of an operating microscope in the anesthetized rats. After the abdominal aortic nerve was cut distally to ensure that the afferent activity was not recorded, the nerve was placed on a pair of silver recording electrodes and was immersed in warm mineral oil. The signals were amplified, passed through a band pass filter (10- to 3 kHz, DAM50-E, World Precision Instruments Inc., Sarasota, FL, USA) and displayed on an oscilloscope. The filtered signal of the nerve activity was rectified, integrated, and collected for display and analysis using a PowerLab 35 Series data acquisition system (AD Instruments, Bella Vista, New South Wales, Australia).

The baroreflex responses^48^ of the Nx, RD and sham groups under anesthesia were elicited with increasing doses of phenylephrine (10 to 30 μg/kg intravenous) to test the rats’ phenylephrine-induced baroreflex responses. The peak bradycardia reflex elicited by phenylephrine was expressed as the pulse period (ms) and plotted against the respective peak increase in SBP for the 3 different doses of phenylephrine in each animal. The slope of the linear regression curve between the increases in pulse period and BP was used to express the baroreflex sensitivity.

### Real-time PCR analysis

Total cellular RNA was isolated from the NTS using an RNAqueous kit (Invitrogen). Aliquots of 1 μg of RNA were reverse transcribed with a SuperScript III first-strand synthesis kit from Invitrogen, according to the manufacturer’s instructions. All primers were synthesized by Operon Technologies (Alameda, CA, USA). The primer sequences for GABA_A_ receptor were 5′-TGTGGCAGAAGATGGGTCAC-3′ for the forward primer and, 5′-ACGGTCGTCACTCCAAAGAC-3′ for the reverse primer and amplified 249 base pairs of the GABA_A_ receptor cDNA fragment. The GABA_B_ receptor mRNA level was determined using 5′-TCACCTCAACGCTGGATGAC-3′ for the forward primer and 5′-GTTGTCAGCATACCACCCGA-3′ for the reverse primer, which amplified 242 base pairs of the GABA_B_ receptor cDNA fragment. The primer sequences for GAD1 were the following: forward primer, 5′-GTGGAAAGCAAAGGGCACTG-3′; and reverse primer, 5′-AGGGCTTTGATCTTGGGAGC-3′; these sequences amplified 234 base pairs of the GAD1 cDNA fragment. The primer sequences for GAD2 were the following: forward primer, 5′-AGCTGGAACCACTGTGTACG-3′; and reverse primer, 5′-ACCAGGAGAGCCGAACATTG-3′; these sequences amplified 222 base pairs of the GAD2 cDNA fragment.

### Western Blot Analysis

The protein extracts were separated on 10% SDS-PAGE gels and then transferred to polyvinylidene difluoride membranes (GE Healthcare, Buckinghamshire, UK). Western blotting^49^ was performed using anti-GABAAR, anti-GAD1 and anti-GAD2 (Proteintech, Chicago, USA), anti-GABABR (Novus, Bangkok, Thailand), and anti-β-actin (Chemicon, Temecula, CA) antibodies. The membranes were subsequently incubated with an HRP-labeled goat anti-rabbit (or mouse) secondary antibody. The membranes were developed using an ECL Plus detection kit (GE Healthcare). The resulting bands were visualized and quantified using a Fuji Film Luminescent Image Analyzer (LAS-4000, Fuji Film Co., Tokyo, Japan) and analyzed using Multi Gauge Ver. 3.0 Image Analysis software (Fuji Film).

### Assay of Cerebrospinal Fluid and Serum GABA and Glutamate Levels

GABA concentrations in the cerebrospinal fluid (CSF) and serum samples were assayed using a highly sensitive, competitive inhibition enzyme immunoassay from Cloud-Clone Corp. (USCN, Hubei province, China). The glutamate concentrations in the CSF and serum samples were assayed using the glutamate dehydrogenase-catalyzed oxidation of glutamate, in which the NADH that is formed reduces a formazan (MTT) reagent (BioAssay Systems, CA, USA).

### GAD Activity Assay

The GAD activity was recorded as previously described^50^. The GAD reagent consisted of 1 g of L-glutamic acid, 0.05 g of bromocresol green (colorimetric indicator), 90 g of NaCl, and 3 ml of Triton X-100 per liter. Twenty-five micrograms of NTS tissue lysate was transferred to a test tube, and 1 ml of GAD reagent was added, mixed immediately, and vortexed vigorously for 30 seconds. The tubes were then incubated in a 35 °C water bath, the change in pH was measured after 1 hour, and the OD at 600 nm was measured at the indicated times.

### Histopathological Examination of the Kidneys and Heart

The kidneys and hearts were immediately fixed in 10% buffered formalin for 24 hours and then embedded in paraffin using standard procedures. The kidneys and hearts were sectioned to a 4-μm thickness for histopathological examinations, and the sections were mounted on microscope slides (Menzel, Bielefeld, Germany) and stored dry at room temperature. Periodic acid-Schiff (PAS)-stained sections of the cortex of the renal tissue were scored in a blinded manner. The mid-chamber of the Elastic Van Gieson-stained (EVG stain) sections of heart tissue were scored in a blinded manner.

To visualize the cellular borders, the fixed tissue was incubated with 1 mg/ml wheat germ agglutinin (WGA) conjugated to Alexa Fluor 555 (Invitrogen) in PBS containing 10 mM sodium azide. The immunofluorescence images were captured on a Carl Zeiss LSM 5 PASCAL laser scanning microscope (Carl Zeiss MicroImaging, Göttingen, Germany). ImageJ software was used to quantify the cross-sectional cell surface area of 25 cells per field in 4 fields along the free wall of the mid-chamber, based on WGA-positive staining.

### Echocardiography

As described previously^51^, we performed echocardiographic examinations using commercially available equipment (Vivid 7; GE Vingmed Ultrasound AS, Horten, Norway) equipped with 10 MHz-phased array transducers. The recordings were stored digitally as two-dimensional sine loops and analyzed using Echo PAC software (GE Vingmed Ultrasound AS).

### General Characteristics of Renal Function and Complete Blood Count

We examined the serum creatinine, blood urea nitrogen (BUN) and urine creatinine and protein levels using a Vitros 350 analyzer (Johnson & Johnson) to assess renal function. We also examined the sodium and potassium levels in the serum and urine of each model using an automatic biochemistry analyzer.

### Statistical Analyses

The group data are expressed as the means ± SEMs. An unpaired t-test (for sham and experimental group comparisons) or one-way analysis of variance (ANOVA) with Scheffe’s post hoc comparison was applied to compare the differences between groups. Two-way ANOVA was conducted to assess the effects of preconditioning and time as well as to examine the interactions between SBP. A value of *P* < 0.05 was considered significant.

## Additional Information

**How to cite this article**: Chen, H.-H. *et al*. Renal Denervation Improves the Baroreflex and GABA System in Chronic Kidney Disease-induced Hypertension. *Sci. Rep.* **6**, 38447; doi: 10.1038/srep38447 (2016).

**Publisher's note:** Springer Nature remains neutral with regard to jurisdictional claims in published maps and institutional affiliations.

## Supplementary Material

Supplementary Tables and Figures

## Figures and Tables

**Table 1:** Renal function and general characteristics of the sham-operated (sham), 5/6 nephrectomized (Nx), and Nx combined renal denervated (RD) WKY rats.

	Sham (n = 7)	Nx (n = 6)	Nx + RD (n = 8)
	Mean ± SEM	Mean ± SEM	Mean ± SEM
serum BUN (mg/dL)	15.10 ± 0.44	172.07 ± 16.14^a^	63.11 ± 9.12^a,b^
serum creatinine (mg/dL)	0.43 ± 0.02	2.46 ± 0.11^a^	1.24 ± 0.15^a,b^
serum sodium (mmol/L)	147.67 ± 0.56	140.38 ± 3.19^a^	137.20 ± 0.66^a^
serum potassium (mmol/L)	5.68 ± 0.35	7.67 ± 0.29^a^	7.41 ± 0.22^a^
urine protein (mg/dL)	14.58 ± 1.67	446.04 ± 76.88^a^	53.32 ± 6.71^a,b^
urine creatinine (mg/dL)	48.05 ± 2.58	7.96 ± 0.75^a^	11.18 ± 1.20^a,b^
UPCR (ratio)	0.31 ± 0.05	62.84 ± 30.43^a^	4.73 ± 2.12^a,b^
urine sodium (mmol/L)	49.98 ± 7.66	27.05 ± 1.54^a^	25.40 ± 2.54^a^
urine potassium (mmol/L)	148.20 ± 6.34	53.76 ± 3.82^a^	60.99 ± 4.09^a^
urine volume (mL)	14.00 ± 0.77	37.13 ± 3.04^a^	32.00 ± 2.3^a^
drink water (mL)	37.17 ± 1.14	55.71 ± 4.13^a^	44.89 ± 3.28^b^

BUN, blood urea nitrogen; UPCR, urine protein and creatinine ratio. The values are presented as the means ± SEM. ^a^*P* < 0.05 versus the sham group, ^b^*P* < 0.05 versus the Nx group.
