# Supplementary material for: Renal Denervation Improves the Baroreflex and GABA System in Chronic Kidney Disease-induced Hypertension
Source: Sci Rep. 2016 Dec 5;6:38447. doi: 10.1038/srep38447 (PMC5137107; doi:10.1038/srep38447)
Supplement: Supplementary Tables and Figures [file srep38447-s1.pdf]

# Renal Denervation Improves the Baroreflex and GABA System in Chronic Kidney Disease-induced Hypertension

Hsin-Hung Chen[1,6,7], Pei-Wen Cheng[1,7], Wen-Yu Ho[8], Pei-Jung Lu[10], Chi-Cheng Lai[2], Yang-Ming Tseng[3], Hua-Chang Fang[4], Gwo-Ching Sun[9], Michael Hsiao[11] , Chun-Peng Liu [5,12]* and Ching-Jiunn Tseng [1,6,13,14]*

[1]Department of Medical Education and Research, [2]Cardiovascular Center, [3]Department of Pathology and Laboratory Medicine, [4]Division of Nephrology, [5]Department of Administration, Kaohsiung Veterans General Hospital, Kaohsiung, Taiwan; [6]Institute of Clinical Medicine, National Yang-Ming University, Taipei, Taiwan; [7]Yuh-Ing Junior College of Health Care & Management, Kaohsiung, Taiwan; [8]Division of General Internal Medicine, Department of Internal Medicine, [9]Department of Anesthesiology, Kaohsiung Medical University Hospital, Kaohsiung Medical University; [10]Graduate Institute of Clinical Medicine, National Cheng-Kung University, Tainan, Taiwan ; [11]Genomics Research Center, Academia Sinica, Taipei, Taiwan; [12]Section of Cardiology, Department of Medcine, Tri-Service General Hospital, National Defense Medical Center, Taipei, Taiwan; [13]Department of Medical Research, China Medical University Hospital, China Medical University, Taichung, Taiwan; [14]Institute of Biomedical Sciences, National Sun Yat-sen University, Kaohsiung, Taiwan

**Correspondence:**

Ching-Jiunn Tseng, MD, PhD

Department of Medical Education and Research, Kaohsiung Veterans General Hospital, No. 386, Ta-Chung 1st Rd., Kaohsiung, 81362, Taiwan

E-mail: cjtseng@vghks.gov.tw; Tel: 886-7-3422121 ext.1505; Fax: 886-7-3468056

or to:

Chun-Peng Liu, MD

Department of Administration, Kaohsiung Veterans General Hospital, No. 386, Ta-Chung 1st Rd., Kaohsiung, 81362, Taiwan

E-mail: cpliu@vghks.gov.tw; Tel: 886-7-3422121 ext. 2011

## Supplementary Tables

**Table S1. Complete blood counts from the sham-operated (sham), 5/6 nephrectomized (Nx), and Nx combined renal denervated (RD) WKY rats**

| | sham (n=7) | Nx (n=6) | Nx+RD (n=6) |
|---|---|---|---|
| | mean SEM | mean SEM | mean SEM |
| RBC ($10^6$/μL) | 10.58 ± 0.20 | 4.54 ± 0.56 [a] | 6.07 ± 0.22 [a, b] |
| Hb (g/dL) | 18.01 ± 0.20 | 8.42 ± 1.00 [a] | 11.08 ± 0.38 [a, b] |
| Hct (%) | 58.99 ± 0.90 | 25.73 ± 3.05 [a] | 34.64 ± 1.24 [a, b] |

RBC, red blood cells; Hb, hemoglobin; Hct, hematocrit. The values are presented as the means ± SEM. [a]$P$<0.05 vs. the sham group, [b]$P$<0.05 vs. the Nx group.

**Table S2: Heart weight and body weight of the sham-operated (sham), 5/6 nephrectomized (Nx), and Nx combined renal denervated (RD) WKY rats**

| | Sham (n=7) | | Nx (n=7) | | Nx+RD(n=10) | |
|---|---|---|---|---|---|---|
| | mean | SEM | mean | SEM | mean | SEM |
| HW (g) | 1.29 ± | 0.04 | 1.24 ± | 0.05 | 1.14 ± | 0.04 [a] |
| BW (g) | 333.67 ± | 5.67 | 181.00 ± | 13.56 [a] | 197.29 ± | 7.73 [a] |
| HW/BW (Ratio) | 0.39 ± | 0.01 | 0.70 ± | 0.05 [a] | 0.58 ± | 0.02 [ab] |

HW: heart weight; BW: body weight. The data are shown as the means ± SEM. [a]$P$<0.05 vs. the sham group, [b]$P$<0.05 vs. the Nx group.

**Table S3: Echocardiographic assessments of the sham-operated (sham), 5/6 nephrectomized (Nx), and Nx combined renal denervated (RD) WKY rats**

| | Sham (n=7) | Nx (n=7) | Nx+RD (n=10) |
|---|---|---|---|
| | mean SEM | mean SEM | mean SEM |
| FS (%) | 38.84 ± 0.94 | 49.24 ± 2.26 [a] | 42.00 ± 1.24 [b] |
| EDV (mL) | 0.81 ± 0.05 | 0.80 ± 0.05 | 0.95 ± 0.11 |
| EF (%) | 74.73 ± 1.12 | 84.57 ± 1.92 [a] | 77.94 ± 1.35 [b] |
| ESV (mL) | 0.21 ± 0.01 | 0.13 ± 0.02 [a] | 0.22 ± 0.03 [b] |
| IVSd (mm) | 9.02 ± 0.36 | 11.68 ± 0.31 [a] | 10.89 ± 0.45 [a] |
| IVSs (mm) | 10.84 ± 0.40 | 13.71 ± 0.70 [a] | 12.78 ± 0.35 [a] |
| LVd Mass (mg) | 89.92 ± 1.37 | 103.08 ± 1.85 [a] | 104.61 ± 3.11 [a] |
| LVIDd (mm) | 70.71 ± 1.47 | 70.42 ± 1.52 | 74.13 ± 3.07 |
| LVIDs (mm) | 43.31 ± 1.09 | 35.98 ± 2.05 [a] | 43.24 ± 2.45 [b] |
| LVPWd (mm) | 9.54 ± 0.19 | 13.09 ± 0.42 [a] | 12.84 ± 0.35 [a] |
| LVPWs (mm) | 11.98 ± 0.28 | 17.10 ± 1.30 [a] | 15.90 ± 0.38 [a] |
| LVs Mass (mg) | 77.55 ± 1.02 | 81.26 ± 1.77 | 84.92 ± 2.06 [a] |
| SV (mL) | 0.60 ± 0.04 | 0.67 ± 0.04 | 0.73 ± 0.08 |
| CO(mm/mim) | 19.76 ± 1.18 | 15.34 ± 1.11 [a] | 18.11 ± 1.21 |
| HR (BPM) | 328.55 ± 8.19 | 228.59 ± 12.20 [a] | 256.66 ± 10.40 [a] |
| R-R interval (ms) | 183.52 ± 4.08 | 266.79 ± 13.37 [a] | 237.71 ± 10.32 [a] |

FS: Fractional shortening; EDV: End diastolic volume; EF: Ejection fraction; d: diastolic; s: systolic; IVS: Interventricular septum; LV: Left ventricular; ID: Internal diameter; PW: posterior wall thickness; SV: stroke volume; CO: cardiac output; HR: heart rate. The data are shown as the means ± SEM. [a]$P$<0.05 vs. the sham group, [b]$P$<0.05 vs. the Nx group.

**Supplementary Figures**

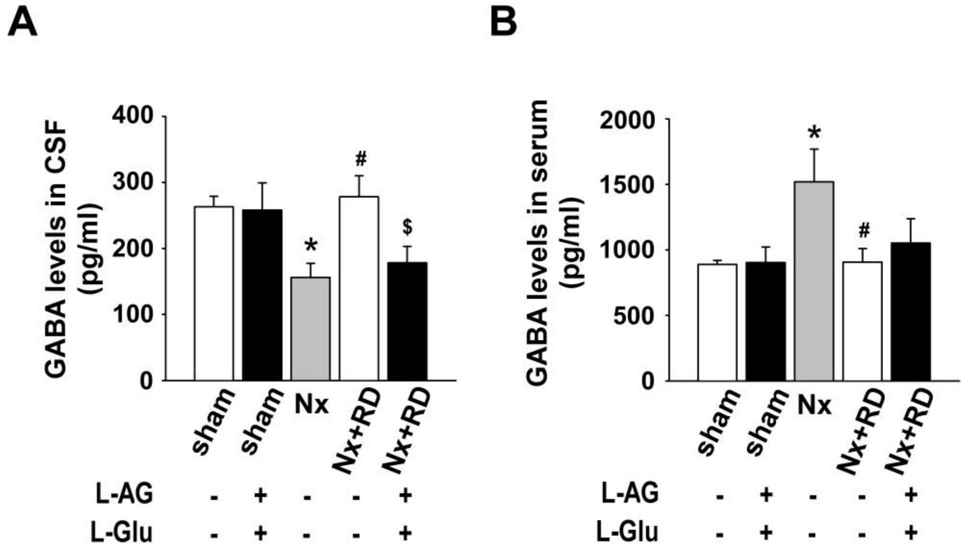


**Supplementary Figure1. The GABA levels are reduced the Nx+RD groups in the CSF after ICV injections of L-AG and L-Glu.** The GABA levels in the CSF and serum in each group were determined using an ELISA kit. The data are shown as the means ± SEM (n=4-6). **P*<0.05 versus the sham group. [#]*P*<0.05 versus the Nx group. [$]*P*<0.05 versus the Nx+RD group.

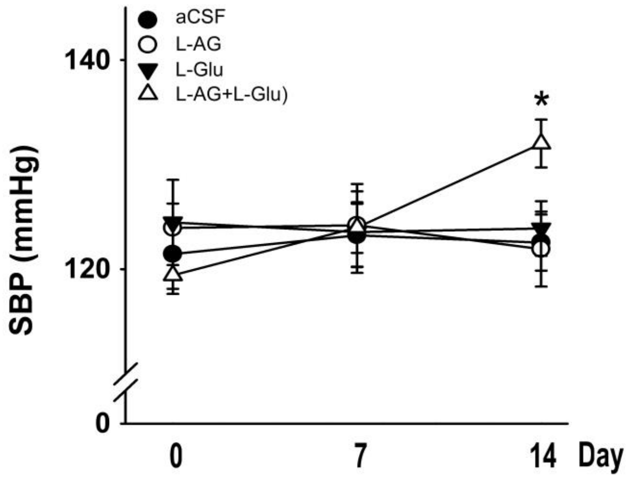


**Supplementary Figure 2. Mimicking the central effect of CKD elevates the BP of the normotensive rats.** ICV injections of LAG and/or L-Glu were administered using a minipump in the non-nephrectomized rats for 2 weeks. The data are shown as the means ± SEM (n=4-6). **P*<0.05 versus the sham group.
